# Brown Algae Polyphenol, a Prolyl Isomerase Pin1 Inhibitor, Prevents Obesity by Inhibiting the Differentiation of Stem Cells into Adipocytes

**DOI:** 10.1371/journal.pone.0168830

**Published:** 2016-12-30

**Authors:** Atsuko Suzuki, Toshiyuki Saeki, Hiroko Ikuji, Chiyoko Uchida, Takafumi Uchida

**Affiliations:** 1 Molecular Enzymology, Department of Molecular Cell Science, Graduate School of Agricultural Science, Tohoku University, 1–1 Amamiya, Tsutsumidori, Aoba, Sendai, Miyagi, Japan; 2 Department of Human Development and Culture, Fukushima University, Kanayagawa 1, Fukushima, Fukushima, Japan; Universidad Pablo de Olavide, SPAIN

## Abstract

**Background:**

While screening for an inhibitor of the peptidyl prolyl cis/trans isomerase, Pin1, we came across a brown algae polyphenol that blocks the differentiation of fibroblasts into adipocytes. However, its effectiveness on the accumulation of fat in the body has never been studied.

**Methodology/Principal Findings:**

Oral administration of brown algae polyphenol to mice fed with a high fat diet, suppressed the increase in fat volume to a level observed in mice fed with a normal diet. We speculate that Pin1 might be required for the differentiation of stem cell to adipocytes. We established wild type (WT) and *Pin1*^*-/-*^ (Pin1-KO) adipose-derived mesenchymal stem cell (ASC) lines and found that WT ASCs differentiate to adipocytes but Pin1-KO ASCs do not.

**Conclusion and Significance:**

Oral administration of brown algae polyphenol, a Pin1 inhibitor, reduced fat buildup in mice. We showed that Pin1 is required for the differentiation of stem cells into adipocytes. We propose that oral intake of brown algae polyphenol is useful for the treatment of obesity.

## Introduction

It has been reported that the phosphorylated Ser/Thr-Pro-specific peptidyl prolyl cis/trans isomerase, Pin1, plays an important role in a variety of diseases [1, 2]. We ascertained the effects of Pin1 on obesity and diabetes, since Pin1 expression is increased by a high-fat diet, which is one of the most common causes of obesity [3–7]. We elucidated that Pin1 plays a critical role in energy generation in the body by enhancing insulin signaling. Pin1 associates with CRTC2, a co-activator of CREB (cAMP-response element-binding protein), and suppresses its transcriptional activity [3]. In addition, Pin1 positively regulates insulin signaling by enhancing insulin receptor substrate-1 (IRS-1), which is a major substrate of this receptor [4]. These results suggest that Pin1 is a promising target molecule for treating obesity and diabetes.

Many of the therapeutics approved to date are either natural products or their derivatives [8–10], and immunosuppressant drugs, Cyclosporin A, and FK506, which target the PPIase subfamilies, cyclophilins, and FK506-binding proteins respectively, are natural products as well [11]. Therefore, we applied the high throughput screening method to screen libraries of natural products including seafood and discovered the Pin1 inhibitor, 974-B, from the edible seaweed, that inhibits differentiation of NIH3T3-L1 cells into adipocytes [12–15].

Here we report that mice fed with a high fat diet in addition to the brown algae polyphenol extract gained significantly less fat than mice fed just with a high fat diet alone. We speculated that the brown algae polyphenol extract might have blocked the differentiation of mesenchymal stem cells into adipocytes. We created WT and Pin1-KO ASC lines from *Pin1*^*+/+*^*; p53*^*-/-*^ and *Pin1*^*-/-*^ *p53*^*-/-*^ mouse fat cells respectively, and examined whether Pin1 is required for differentiation of mesenchymal stem cells into adipose cells.

## Results

**Fig 1:**
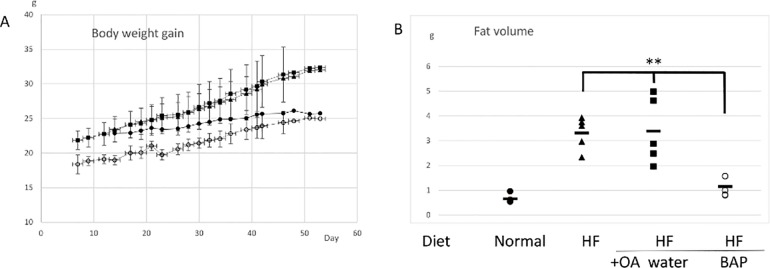
**A) Comparison of weights of mice** fed with a normal diet, a high fat diet, a high fat diet plus water, and a high fat diet plus brown algae polyphenol. C57BL/6j male mice at 4-weeks of age (n = 5 each) were fed with a normal diet (closed circle), a high fat diet (triangle), a high fat diet with oral administration of water (closed square), and a high fat diet with oral administration of brown algae polyphenol (open circle) (means ± SD). Oral administration of brown algae polyphenol or water was started after the mice were provided water freely and no food for a day. 10 μM of brown algae polyphenol was provided with the high fat diet for 8 weeks. B) **Comparison of fat volume in mice** (n = 5) fed with a normal diet (closed circle), a high fat diet (triangle), a high fat diet with oral administration of water (closed square), and a high fat diet with oral administration of brown algae polyphenol (open circle). Water for group3 and brown algae polyphenol for group4 were administered orally. The data were analyzed by 1-way Anova followed by Bonferroni post Hoc test. **P<0.01.

Four-week old mice fed with a high fat diet showed increased body weight compared to the mice fed with a normal diet. Oral administration of brown algae polyphenol remarkably suppressed gaining weight, even though the mice were fed with a high fat diet. The weight gain of the mice fed with a high fat diet with brown algae polyphenol was almost the same as that of the mice fed with a normal diet (Fig 1A)(S1A Fig). Oral administration itself did not stress the mice, since oral administration of water did not affect weight.

We found the significant difference between the mice fed with the high fat diet plus brown algae extract and the mice fed with the high fat diet plus water with regards to subcutaneous and visceral fat volume (Fig 1B)(S1B Fig). These results clearly showed that the weight loss caused by administration of brown algae polyphenol correlates with the loss of fat in mice.

**Fig 2:**
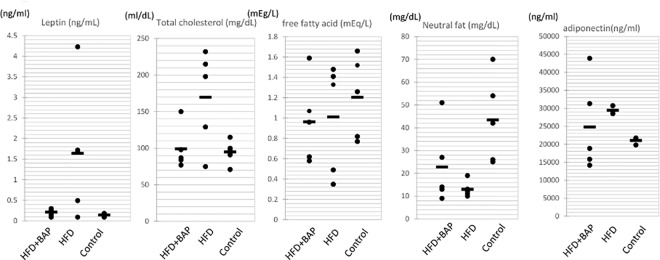
Comparison of obesity marker levels in sera between mouse groups (n = 5 each). Leptin (ng/ml), total cholesterol (mg/dl), free fatty acids (mEq/L), neutral fat (mg/dl), and adiponectin (ng/ml) in the serum of each mouse, 1: high fat diet + brown algae polyphenol, 2: high fat diet, 3: normal diet.

The levels of molecules, such as leptin, total cholesterol, free fatty acids, neutral fat, and adiponectin were compared among the mice fed with a normal diet, the mice fed with a high fat diet, and the mice fed with a high fat diet with brown algae polyphenol. The levels of leptin and cholesterol in the mice fed with the high fat diet plus brown algae polyphenol were similar to that in the mice fed with the normal diet. The levels of leptin and cholesterol in the mice fed with the high fat diet were higher than that in the mice fed with the normal diet (Fig 2)(S2 Fig). These results show that brown algae polyphenol changed the levels of obesity marker molecules into that of the mice fed with a normal diet.

**Fig 3:**
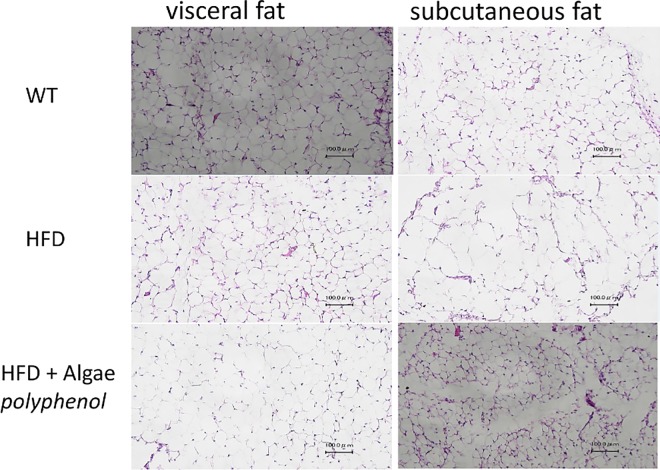
Paraffin-embedded sections of visceral and subcutaneous fat tissue from mice fed with a normal diet, high fat diet (HFD) and high fat diet plus brown algae polyphenol (HFD + BAP) were stained with hematoxylin and eosin.

The histopathology of fat tissues in these mice was compared. The sizes of adipose cells in the mice fed with the high fat diet were larger than that in the mice fed with a normal diet. The sizes and the shapes of adipocytes in the mice fed with the high fat diet plus brown algae polyphenol were similar to those in the mice fed with the normal diet (Fig 3).

**Fig 4:**
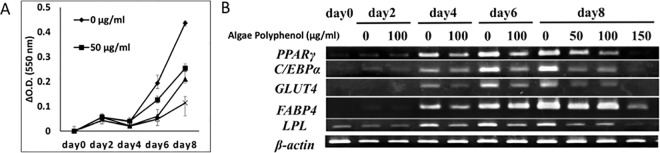
Effect of brown algae polyphenol on the differentiation of NIH3T3-L1 cells to adipocytes. **A) The amount of the intracellular lipid stained with oil red O.** NIH3T3-L1 cells were cultured in DMEM containing 0.5 mM 3-isobutyl-1-methylxanthine, 1 μM dexamethasone, and 1.7 μM insulin with 0 (diamond), 50 (square), 100 (triangle), and 150 μg/ml (x) brown algae polyphenol for 0–8 days. NIH3T3-L1 cells were treated with 4% paraformaldehyde and 60% 2-propanol and then stained with Oil Red O. The amount of oil red O extracted from the cells was determined three times by measuring absorbance at 550 nm (means ± SEM). **B) PCR analysis of adipocyte biomarker** mRNA levels. NIH3T3-L1 cells were cultured with 0, 50, 100, and 150 μg/ml of brown algae polyphenol for 0–8 days. The mRNA levels of PPARγ, C/EBPα, Glut4, FABP4, and LPL in these cells were compared by PCR.

In order to investigate the mechanism underlying the functions of brown algae polyphenol, we examined the effect of brown algae polyphenol on the differentiation of NIH3T3-L1 cells into the adipocyte-like cells. As shown in Fig 4A (S3 Fig), lipid accumulation in the NIH3T3-L1 cells was suppressed by the treatment with the differentiation reagent containing brown algae polyphenol. The levels of adipocyte biomarkers, such as PPARγ, C/EBPα, GLUT4, FABP4, and LPL were examined by PCR. All biomarker levels were activated to a weaker extent in the NIH3T3-L1 cells treated with the differentiation reagent containing brown algae polyphenol than that in the NIH3T3-L1 cells treated with the differentiation reagent alone (Fig 4B).

**Fig 5:**
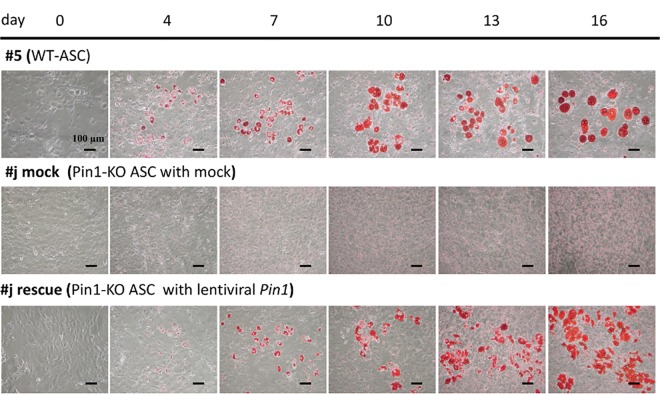
Comparison of ASC differentiation to adipocytes. Comparison of adipocyte differentiation between the WT (*Pin1*^*+/+*^*; p53*^*-/-*^) ASC and Pin1-KO (*Pin1*^*-/-*^*; p53*^*-/-*^) ASC, and Pin1-KO ASC rescued with the lentiviral Pin1 cDNA. The wild type ASC, the Pin1-KO ASC- infected with Mock and the Pin1-KO ASC- infected with lentiviral Pin1 cDNA were cultured in DMEM containing 0.5 mM 3-isobutyl-1-methylxanthine, 1 μM dexamethasone, and 1.7 μM insulin for 0–16 days. ASCs were treated with 4% paraformaldehyde and 60% 2-propanol, and then stained with Oil Red O. The images of the oil red O-stained cells on 0, 4, 7, 10, 13, and 16 days after treatment with the differentiation reagent are shown.

**Fig 6:**
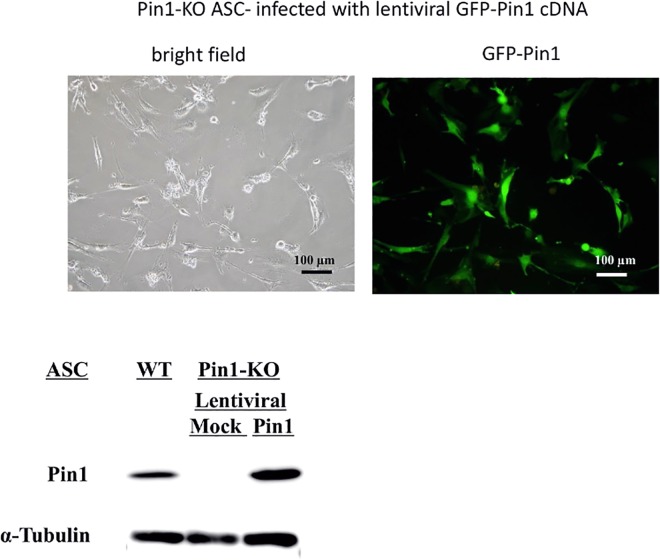
Pin1-KO ASC- transfected with Lentiviral Pin1 cDNA. Transfection was verified by observing GFP with fluorescence microscope and the western blot analysis of Pin1 and α-tubulin (1st: anti-h/m Pin1 antibody (Santa Cruz), anti-α-tubulin antibody (SIGMA), 2nd anti-mouse IgG HRP-linked antibody (Cell Signaling), All antibodies were diluted to 1/1000).

**Fig 7:**
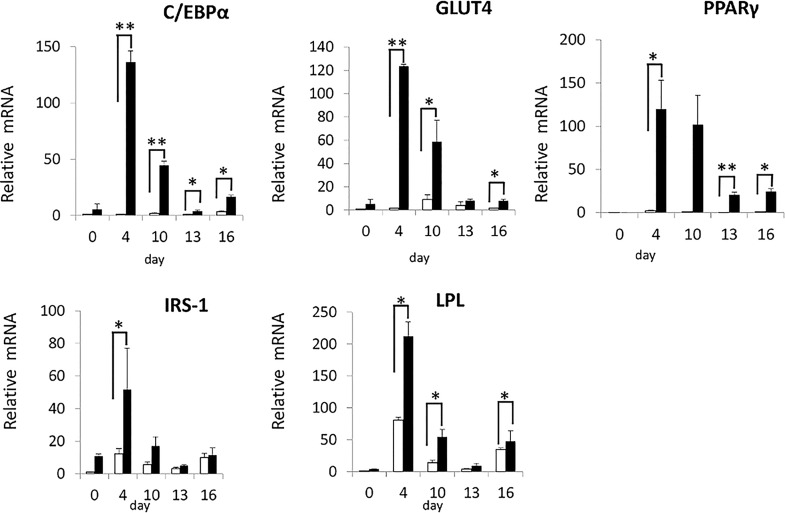
Real time PCR analysis of adipocyte biomarkers. The mRNA levels of adipocyte biomarker molecules, such as C/EBPα, Glut4, PPARγ, IRS1, and LPL in the WT and Pin1-KO ASC treated with the differentiation reagent were quantitatively measured with real time PCR three times using the primers shown in Table 1 (means ± SEM). **p<0.01, * p<0.05.

We then examined the effect of Pin1 on the differentiation of stem cells to adipocytes. We established a Pin1-deficient mesenchymal stem cell line as well as the wild type mesenchymal stem cell line. These lines were established from p53 deficient mice because depletion of p53 is required for immortalization of mesenchymal stem cells [16]. We named these lines as WT (*Pin1*^*+/+*^*; p53*^*-/-*^) and Pin1-KO (*Pin1*^*-/-*^*; p53*^*-/-*^) ASC. Differentiation of ASC lines was induced by a reagent containing 3- isobutyl-1-methylxanthine, dexamethasone, and insulin, and cultured for eight days. Differentiated adipocytes were detected by the amounts of lipid droplets in the cells, upon staining with oil red O. WT ASC showed increased fat droplets and pigments in the cells after 8 days, but Pin1-KO ASC did not show the lipid droplets. The rescued Pin1-KO ASC, where Pin1 expression was restored by lentiviral Pin1 cDNA infection, differentiated into adipocytes in a manner similar to the WT ASC (Figs 5 and 6). Differentiation of ASC to adipocytes was also examined by the expression levels of adipocyte biomarker mRNAs such as C/EBPα, GLUT4, PPARγ, IRS-1, and LPL. These marker RNA levels were increased within four days after treatment with the differentiation medium in WT ASC but not in Pin1-KO ASC (Fig 7)(S4 Fig).

## Discussion

We found that gain in body weight by a high fat diet is suppressed by oral administration of brown algae polyphenol. Decrease in fat volume of mice fed with a high fat diet to the same level as that of fat volume in the mice fed with a normal diet was statistically significant. On the other hand, brown algae polyphenol maintained the levels of adipocyte markers to levels similar to those in mice fed with a normal diet, including adiponectin levels, which is a good cytokine for diabetes. These results suggest that brown algae polyphenol reduces the excessive generation of adipocytes without reducing adipocyte activity.

The histopathological analysis of fat tissues from these mice strongly supports that brown algae polyphenol decreases the number of adipocytes. These results suggested that the differentiation of adipocyte stem cells was suppressed by brown algae polyphenol. We chose the NIH3T3-L1 cells to examine the effect of brown algae polyphenol on adipocytic differentiation, because NIH3T3-L1 is the most popular cell line for the evaluation of cellular differentiation to adipocytes. We reported that the siRNA against Pin1 inhibited differentiation of NIH3T3-L1 cells and the inhibitory effect of Pin1 depletion was further confirmed by using two known Pin1 inhibitors, juglone and PiB (diethyl- 1,3,6,8- tetrahydro- 1,3,6,8- tetraoxobenzol—phenanthroline- 2,7-diacetate) (7). Recently it was reported that oral administration of juglone, a Pin1 inhibitor, with high-fat diet in rats suppressed gaining weights [17], which suggests that brown algae polyphenol suppressed gaining weights as a result of inhibiting Pin1. In this report, we showed that brown algae polyphenol suppresses differentiation of stem cells into adipocytes by using the ASC lines that are much the same as natural preadipocytes.

In order to confirm this function of Pin1 in cells, we established WT and Pin1-KO ASC lines. Comparing the differentiation of these lines to adipocytes proved that Pin1 is required for differentiation of ASC to adipocytes. Differentiation of cells to adipocytes is mainly regulated by two transcription factors, C/EBPα and PPARγ [18]. The levels of these factors were upregulated in WT ASC but not Pin1-KO ASC. In addition to these factors, other marker molecules, such as LPL [19], IRS-1 [4], and GLUT4 [20] were decreased in Pin1-KO ASC. It is reported that Pin1 is required for culturing pluripotent stem cells while maintaining an undifferentiated state by stabilizing Nanog and Oct4 [21, 22]. Our results suggest that Pin1 is also required for the differentiation of mesenchymal stem cells to adipocytes. Taken together we speculate that Pin1 regulates stem cell differentiation by affecting several kinds of molecules that appear at different stages.

Here we report that Pin1 is required for initiating stem cell differentiation smoothly once differentiation is induced in the cell. We showed that dietary intake of brown algae polyphenol suppresses ASC differentiation to adipocytes and prevents obesity.

## Materials and Methods

### Animals

Our study was approved by the Tohoku University Animal Use and Care Committee. C57BL/6j mice (3 weeks) were purchased from SLC Inc. (Hamamatsu, Japan), and given free access to food and water. High fat diet, 20–25 g/week of HFD-60 (oriental Kobo), was administered to 4-week- old male mic (n = 5), with body weight being measurements taken every two days. C57BL/6j male mice at 4-weeks of age (n = 5 each) were fed with a normal diet, a high fat diet and a high fat diet with brown algae polyphenol. The brown algae polyphenol (70% purity) was kindly provided by Nagase Co (14) and dissolved it to water (10 μM). Brown algae polyphenol solution was administrated orally with medication syringe. The administration was started after the mice were provided water freely and no food for a day. 10 μM of brown algae polyphenol was provided with the high fat diet for 8 weeks. The same treatment just with water was performed as the control. As shown in the previous paper, the weight of fat tissue, such as buttock fat tissue, which represents subcutaneous fat, and genital fat tissue, which represents visceral fat was measured. Comparison of fat volume in mice fed with a normal diet, a high fat diet, a high fat diet plus water, and a high fat diet plus brown algae polyphenol. The average fat weight of each group is shown by the bars.

Our study was approved by the Tohoku University Animal Use and Care Committee. Mice were cared in SPF condition and fed with certified diet MF (Oriental yeast Co., LTD). The microbial test was done every three months, but we examined the mouse condition every day during experiment. No mice became ill or died. We determined the condition of mice from their activities and also the results of microbial monitoring. The method of euthanasia for all animals utilized in this research was cervical dislocation.

### Statistical analysis

Values are reported as means ± SD (Fig 1A) or means ± SEM (other figures). Statistical analysis of differences between mean values was carried out by 1-way Anova followed by Bonferroni post Hoc test for Fig 1B and Student’s t tests for Fig 7.

Adipose-Derived Mesenchymal Stem Cells (ASC)

The WT (*Pin1*^*+/+*^*; p53*^*-/-*^) and the Pin1-KO (*Pin1*^*-/-*^*; p53*^*-/-*^) ASC lines were prepared from *Pin1*^*+/+*^*; p53*^*-/-*^ and *Pin1*^*-/-*^*; p53*^*-/-*^ mice respectively as previously described [16].

### Cell culture and induction of adipocyte differentiation

The differentiation of NIH3T3-L1 cells and ASCs to adipocytes were performed as written in the previous report (15). These cells (4 × 104 per well) were cultured in 24-well plates coated with type I collagen until 100% confluency. The cells were cultured in Dulbecco’s Modified Eagle Medium (DMEM) containing 0.5 mM 3-isobutyl-1-methylxanthine (Nacalai tesque), 1 μM dexamethasone (Sigma), and 1.7 μM insulin (Wako) with brown algae polyphenol (0–150 μg/ml) for a span of 16 days.

### Oil Red O staining for cultured cells

ASCs and NIH3T3-L1 cells were treated with 4% paraformaldehyde and 60% 2-propanol and then stained with Oil Red O (Sigma). The stained cells were observed under a BZ-8100 microscope (Keyence) and the quantity of Oil Red O-extracted from cells was determined by measuring absorbance at 550 nm using the infinite TM M200 (TECAN).

### Lentiviral-Pin1 cDNA

Pin1-KO ASC were transfected with lentiviral Pin1 cDNA that was prepared as the following. HEK293T cells were transfected with the packaging plasmids and the Pin1 expression vector, pCDH- CMV-Pin1-EF1-copGFP or pCDH-CMV-MCS-EF1-copGFP lentiviral vector. These plasmids were kindly provided by Hiroyuki Miyoshi, RIKEN. 3 μg of pCDH-CMV-Pin1-EF1-copGFP, pCDH-CMV- MCS-EF1-copGFP, pCMV-VSVG-RSV-REV, and pCAG-HIV-gp solution was mixed with polyethylenimine solution (18 μl in 800ul DMEM) at 25°C for 20 min, and added the solution into the dish where HEK293T was cultured to 80% confluent. The cell Pin1-KO ASC were transfected with lentiviral Pin1 cDNA that was prepared as the following. HEK293T cells were transfected with the packaging plasmids and the Pin1 expression vector, pCDH- CMV-Pin1-EF1-copGFP or pCDH-CMV-MCS-EF1-copGFP lentiviral vector. These plasmids were kindly provided by Hiroyuki Miyoshi, RIKEN. 3 μg of pCDH-CMV-Pin1-EF1-copGFP, pCDH-CMV- MCS-EF1-copGFP, pCMV-VSVG-RSV-REV, and pCAG-HIV-gp solution was mixed with polyethylenimine solution (18 μl in 800ul DMEM) at 25°C for 20 min, and added the solution into the dish where HEK293T was cultured to 80% confluent. The cells with the plasmids were incubated in a CO_2_ incubator at 37°C for 24 hours. The packaging was verified by observing fluorescence microscope BZ-8100 (Keyence) and the cells were incubated for 48 hours. The supernatant of the medium was filtrated with 0.45 μm membrane filter (Millipore) and then the virus was concentrated by adding 1/3 volume of the mixture containing 32% PEG6000,0.4 M NaCl, 0.04 M HEPES. After 24 hours, the viral solution was centrifuged and used for infection to ASCs. Transfection was confirmed by the presence of GFP fluorescence in the cell and western blotting for the presence of Pin1 in the cell lysate.

### Western blot

Pin1-KO ASC- transfected with Lentiviral Pin1 cDNA. Transfection was verified by observing GFP with fluorescence microscope and the western blot analysis of Pin1 and α-tubulin (1st: anti-h/m Pin1 antibody (Santa Cruz), anti-α-tubulin antibody (SIGMA), 2nd anti-mouse IgG HRP-linked antibody (Cell Signaling), All antibodies were diluted to 1/1000).

### Real time PCR

WT and Pin1-KO ASC were treated with the differentiation reagent as described in Cell culture and induction of adipocyte differentiation. RNA was extracted from these cells after 0, 4, 10, 13, and 16 days of treatment using the Trizol reagent (Life Technologies), and cDNAs were synthesized using the Prime Script 1st Strand cDNA Synthesis Kit (Takara Bio). Real Time PCR analysis of C/EBPα, Glut4, PPAR γ, IRS1, and LPL was performed using the primers shown in Table 1 (PTC200 Peltier Thermal Cycler; MJ Research).

**Table 1:** Primer List for the Real-time PCR

PPARγ forward	5’-TGCTGTTATGGGTGAAACTCTG -3’
PPARγ reverse	5’-CTGTGTCAACCATGGTAATTTCTT-3’
C/EBPα forward	5’-CGCAAGAGCCGAGATAAAGC-3’
C/EBPα reverse	5’-CGGTCATTGTCACTGGTCAACT-3’
GLUT4 forward	5’- CTGTAACTTCATTGTCGGCATGG-3’
GLUT4 reverse	5’- AGGCAGCTGAGATCTGGTCAAAC-3’
LPL forward	5’- GGTCAGACTTCCTGCTACGC-3’
LPL reverse	5’- GCCCAGCAACATTATCCAGT-3’
IRS-1 forward	5’- TCCAACCTCCACACTGATGA-3’
IRS-1 reverse	5’- ATCTGCTGTGGGGCAGATAC-3’
β-actin forward	5’- GGCTGTATTCCCCTCCATCG-3’
β-actin reverse	5’- CCAGTTGGTAACAATGCCATGT-3’

## Supporting Information

S1 Fig Raw data for Fig 1A and Fig 1B. Data for mouse weights (A) and fat volume (B).
(XLSX)

S2 Fig Raw data for Fig 2. Data for obesity marker molecules, such as neutral fat, free fatty acid, total cholesterol, leptin.
(XLSX)

S3 Fig Raw data for Fig 4A. Data for intra cellular lipid stained with oil red O.
(XLSX)

S4 Fig Raw data for Fig 7. Data for real time PCR of C/EBPα, Glut4, PPARγ, IRS1, and LPL.
(XLSX)

## Abbreviations

ASC: adipose-derived mesenchymal stem cel
WT: wild type
Pin1-KO: Pin1 knockout or *Pin1*^*-/-*^
WT ASC: *Pin1*^*+/+*^*; p53*^*-/-*^ ASC
Pin1-KO ASC: *Pin1*^*-/-*^*; p53*^*-/-*^ ASC
IRS-1: insulin receptor substrate-1
HFD: high fat diet
BAP: brown algae polyphenol
